# Stochastic assembly in a subtropical forest chronosequence: evidence from contrasting changes of species, phylogenetic and functional dissimilarity over succession

**DOI:** 10.1038/srep32596

**Published:** 2016-09-07

**Authors:** Xiangcheng Mi, Nathan G. Swenson, Qi Jia, Mide Rao, Gang Feng, Haibao Ren, Daniel P. Bebber, Keping Ma

**Affiliations:** 1 State Key Laboratory of Vegetation and Environmental Change, Institute of Botany, Chinese Academy of Sciences, Beijing, 100093, China; 2 Department of Biology, University of Maryland, College Park, Maryland, 20742 USA; 3 College of Chemistry and Life Science, Zhejiang Normal University, Jinhua, Zhejiang 321004, China; 4 Earthwatch Institute, Mayfield House, 256 Banbury Road, Oxford, OX2 7DE, United Kingdom

## Abstract

Deterministic and stochastic processes jointly determine the community dynamics of forest succession. However, it has been widely held in previous studies that deterministic processes dominate forest succession. Furthermore, inference of mechanisms for community assembly may be misleading if based on a single axis of diversity alone. In this study, we evaluated the relative roles of deterministic and stochastic processes along a disturbance gradient by integrating species, functional, and phylogenetic beta diversity in a subtropical forest chronosequence in Southeastern China. We found a general pattern of increasing species turnover, but little-to-no change in phylogenetic and functional turnover over succession at two spatial scales. Meanwhile, the phylogenetic and functional beta diversity were not significantly different from random expectation. This result suggested a dominance of stochastic assembly, contrary to the general expectation that deterministic processes dominate forest succession. On the other hand, we found significant interactions of environment and disturbance and limited evidence for significant deviations of phylogenetic or functional turnover from random expectations for different size classes. This result provided weak evidence of deterministic processes over succession. Stochastic assembly of forest succession suggests that post-disturbance restoration may be largely unpredictable and difficult to control in subtropical forests.

A major challenge in studies of community assembly is to understand the relative importance of deterministic versus stochastic processes determining species composition^1^,^2^,^3^. Niche-based equilibrium theory assumes that local community dynamics are shaped by deterministic processes dictated by the relationship between species-specific traits and local abiotic and biotic factors^1^,^3^. In contrast, a stochastic view suggests that community dynamics are determined solely by demographic stochasticity and dispersal limitation^2^,^4^. Hitherto studies on the relative importance of these two sets of processes in forest ecology have mainly focused on mature species-rich forests rather than secondary forests. However, the directly observable rapid community dynamics and striking changes of the forest structure in disturbed, successional communities provide a useful laboratory for studying community assembly^5^,^6^.

Species beta diversity or species turnover measures the rate of community compositional changes between two or more locations^7^,^8^. Exploring patterns of species beta diversity can provide considerable insights into the relative roles of deterministic and stochastic processes during secondary forest succession. For example, based on Clement’s^9^ concept of succession, the deterministic view postulates that forest in all locations converge on a single optimal assemblage over succession. The convergence may be resulted from decreasing variation in environmental conditions among locations such as relatively homogeneous shade after canopy closure^4^,^10^. Thus, the deterministic view assumes a trend of decreasing species beta diversity over succession^11^,^12^. In contrast, the stochastic view proposes that dispersal limitation and demographical stochasticity across locations would result in increasingly divergent species composition, and suggests an increase in species beta diversity with succession^2^. Christensen and Peet^11^ tested these predictions by examining species beta diversity and have found that in forests of five age-classes, the mature hardwood stands had the highest beta diversity while young pine stands had the lowest beta diversity, thus failing to support Clement’s deterministic view^9^.

However, despite these predictions, deterministic and stochastic processes may potentially produce similar patterns of species beta diversity over succession^4^,^13^. For example, increasing species beta diversity created by stochastic processes may also arise from deterministic processes such as increasing variation in environmental conditions across locations. Severe disturbances such as forest clearing may homogenize environmental conditions like light and soil properties. The post-disturbance recovery of variation in environmental conditions such as understory light and soil nutrient levels, may lead to increasing species beta diversity when species with different traits colonize environmentally heterogeneous locations. The same species beta diversity pattern could be created simultaneously by both stochastic and deterministic processes. Thus, it is difficult to discern the relative roles of deterministic and stochastic processes, by solely using a species-based beta diversity approach.

Considering functional beta diversity may provide complementary information to species beta diversity, it may help to overcome this limitation. Environmental conditions determine the available niches and the functional composition that can fill them, thus community assembly is expected to be deterministic in functional composition. Moreover, disturbance regimes, i.e., different levels of disturbance intensity and frequency create various levels of variation in environmental conditions among locations and initial community composition^14^. Therefore, functional beta diversity should decrease (or increase) when variation in initial environmental conditions decrease (or increase) over time or along a disturbance gradient. In contrast, functional beta diversity should not change significantly if late-arriving species have similar traits with early-arriving species (priority effect) or community dynamics and species performance are not governed by species traits. Analyses integrating species, functional and phylogenetic beta diversity approaches may provide ecologists better understanding of multiple facets of community assembly over succession. For example, Fukami *et al*.^15^ experimentally demonstrated in a 9-year study that a decline in functional beta diversity and an constant species beta diversity over succession may arise from simultaneous operations of deterministic processes and priority effect.

Functional beta diversity is based on a limited set of measurable traits, whereas phylogenetic relatedness approximates multivariate trait similarity of species^16^. Therefore, phylogenetic beta diversity may potentially provide additional insight by synthesizing a greater amount of information than functional beta diversity. However, inferences of community assembly from community phylogenetic structures strongly depend on the niche conservatism assumption that closely related species have similar niches. If traits are not phylogenetically conserved, it is difficult to understand the community assembly processes from community phylogenetic structures.

In this study, we aimed to evaluate the relative roles of deterministic and stochastic processes in forest succession by using multiple axes of beta diversity. We used a dataset from nine 1-ha stem-mapped plots along a disturbance gradient in a subtropical forest chronosequence in southeastern China. As found in recent studies, strong environmental filtering governs forest succession in early stage, while interspecific competitions dictate community assembly in late stage^17^,^18^,^19^. We thus hypothesized that deterministic processes dominate the subtropical forest succession. On the other hand, the relative importance of local processes strongly depends on the chosen scales^20^,^21^. For example, neighborhood interaction operates at smaller spatial scales than environmental filtering^22^. Thus we tested our hypothesis at spatial scales of 10 × 10 m and 20 × 20 m.

## Results

### Phylogenetic signal in traits

The phylogenetic signal of six examined traits were quantified using Blomberg’s *K*^23^. Six traits had a weak phylogenetic signal with *K* values of less than one (Table B1), ranging from 0.105 to 0.441. However, all traits, except leaf area, had *K* values significantly higher than expected by chance, indicating a weak but significant phylogenetic signal. The significant phylogenetic signal in traits suggested that patterns of phylogenetic turnover may mirror the patterns of functional turnover.

### The effect of disturbance regimes on spatial turnover of diversity for all stems

**Figure 1:**
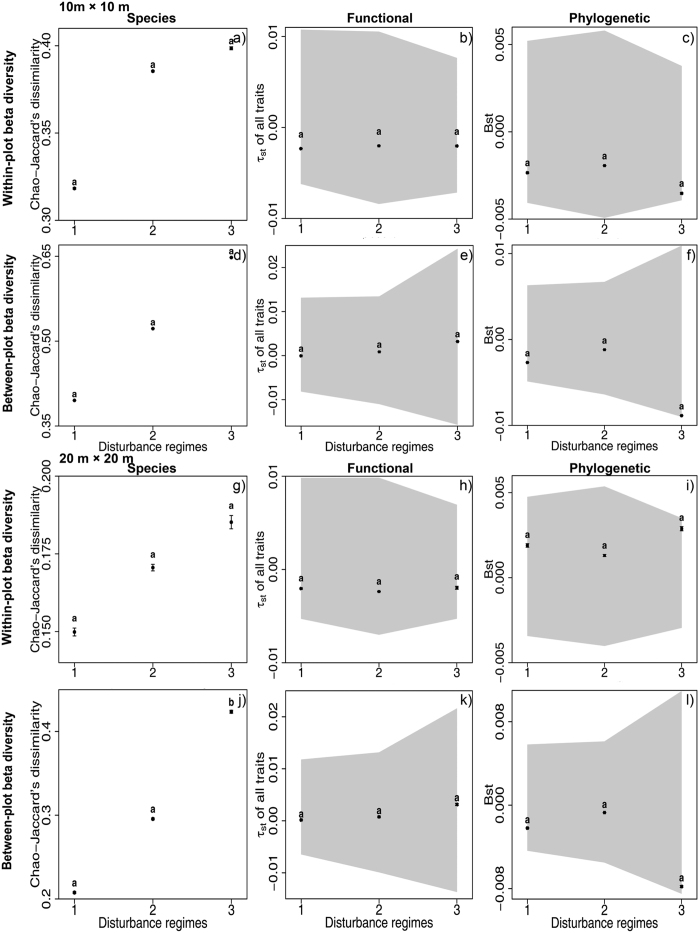
Species, functional and phylogenetic beta diversity for all subplot pairs (black square, mean ± SE) within different disturbance regimes at within- and between-plot level and scales of 10 × 10 m and 20 × 20 m. (**a,g**) are species beta diversity at within-plot level, (**b,h**) for functional beta diversity at within-plot level, (**c,i**) for phylogenetic beta diversity at within-plot level, while (**d,j**) are species beta diversity at between-plot level, (**e,k**) for functional beta diversity at between-plot level, (**f,l**) for phylogenetic beta diversity at between-plot level. Letters indicate significant differences (P < 0.05) between disturbance regimes which was calculated by multiple comparison. The grey-shaded area represents the 95%-confidence interval for *B*_*st*_ and *τ*_*st*_ values from the 999 random communities. *B*_*st*_ and *τ*_*st*_ values inside the interval indicate phylogenetic or functional randomness, and *B*_*st*_ and *τ*_*st*_ values outside the interval indicate the significant phylogenetic or functional clustering or dispersion. Disturbance regimes: 1. young secondary forest: clear-cutting 50 years ago and selective cutting 20 years ago, 2. old secondary forest: clear-cutting 50 years ago, 3. old growth forest: without human disturbance more than 100 years.

**Figure 2:**
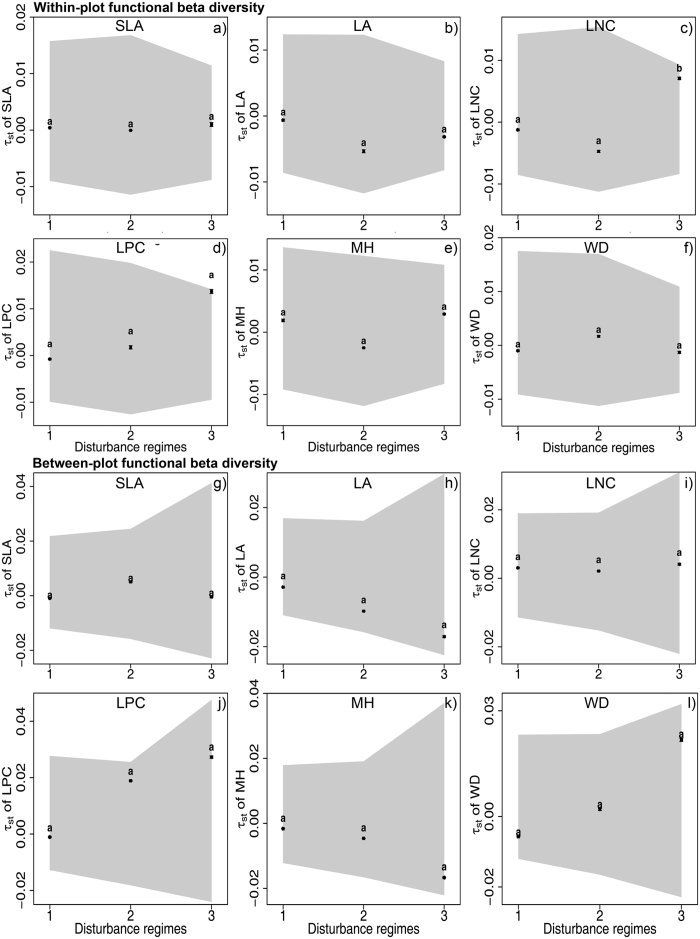
Functional beta diversity of individual traits for all subplot pairs (black square, mean ± SE) within different disturbance regimes at within-plot and between-plot levels and at a scale of 20 × 20 m. Letters indicate significant differences (P < 0.05) between disturbance regimes which was calculated by multiple comparison. The grey-shaded area represents the 95%-confidence interval for the *τ*_*st*_ values from the 999 random communities. *τ*_*st*_ values inside the interval indicate phylogenetic or functional randomness, and *τ*_*st*_ values outside the interval indicate the significant phylogenetic or functional clustering or dispersion. SLA = specific leaf area, LA = leaf area, WD = wood density, LPC = leaf phosphorus content, LNC = leaf nitrogen content, MH = maximum height. Disturbance regimes: 1. young secondary forest: clear-cutting 50 years ago and selective cutting 20 years ago, 2. old secondary forest: clear-cutting 50 years ago, 3. old growth forest: without human disturbance more than 100 years.

In this study, linear mixed models were used to determine the effects of disturbance regime on three axes of turnover of diversity. Species, phylogenetic and functional turnover were quantified by Chao-Jaccard dissimilarity, *τ*_*st*_ and *B*_*st*_, respectively. Subsequently, the turnover of three axes of diversity were plotted against disturbance regime (Figs 1 and 2) after controlling for the effect of habitat difference and other potentially important factors. The residuals of all linear mixed models had no significant spatial autocorrelation (illustrated in Fig. C1), suggesting that the spatial structure in the data had been accounted for in the models.

At both scales of 10 × 10 m and 20 × 20 m, we detected increasing trends in species turnover with succession at within-plot or between-plot levels (Fig. 1a,d,g,j), one of which was significant (Fig. 1j). In contrast, functional and phylogenetic turnover (*τ*_*st*_ and *B*_*st*_) of all traits showed no significant trends over succession at both within-plot and between-plot level (Fig. 1b,c,e,f,h,i,k,l). Moreover, *τ*_*st*_ and *B*_*st*_ values of all traits fall inside the 95%-confidence interval of random expectations (Fig. 1b,c,e,f,h,i,k,l), indicating that observed *τ*_*st*_ and *B*_*st*_ values of all traits were not significantly different from random expectations. Despite of insignificant effect of disturbance regime on *τ*_*st*_ and *B*_*st*_, we found significant interaction terms between disturbance regimes and distance of habitat conditions in most phylogenetic and functional turnover models (Tables F1–F2). Functional turnover (*τ*_*st*_) of individual traits showed consistent patterns with that of all traits at two spatial scales (Figs 2, D1).

### The effect of disturbance regimes on spatial turnover of diversity for different size classes

The analyses of three axes of beta diversity for stems of different size classes revealed more details: at both scales of 10 × 10 m and 20 × 20 m, species turnover also tended to show increasing trends (Figs E1a–c, j–l, E2a–c, j–l) for three size classes over time at both within- and between-plot levels, some of which were significant (Figs E1a–c, l).

At a scale of 20 × 20 m, we detected no significant trends of *B*_*st*_ and *τ*_*st*_ along disturbance regimes (Fig. E1d–g,m,o,r) for most dbh size classes. Meanwhile, we found that *B*_*st*_ and *τ*_*st*_ values of all traits fall inside the 95%-confidence interval of random expectations (Fig. E1d–g,m,o,r) for most dbh size classes, indicating that observed *B*_*st*_ and *τ*_*st*_ values were not significant from random expectations. Exceptions included *B*_*st*_ for cohorts of 5–10 cm dbh at within-plot level and 1–5 cm and 5–10 cm dbh classes at between-plot level and *τ*_*st*_ of all traits for cohorts of 5–10 cm dbh class at between-plot level, which displayed significant increasing trends (Fig. E1h,n,p,q), and *B*_*st*_ for cohort of greater than 10 cm dbh at within-plot level which displayed significant decreasing trend (Fig. E1i). On the other hand, we detected significant interaction terms between disturbance regimes and distance of habitat conditions in most phylogenetic and functional dissimilarity models (not shown) for three size classes at the scale of 20 × 20 m. We found similar patterns of *B*_*st*_ and *τ*_*st*_ of all traits for cohorts of all size classes at a scale of 10 × 10 m (Fig. E2).

## Discussion

### The evidence of stochastic processes

At the spatial scale of 10 × 10 m and 20 × 20 m, species turnover showed overall increasing trends over succession (Fig. 1a,d,g,j) for all trees and three size classes, some of which were significant. Functional and phylogenetic turnover displayed little-to-no changes over succession (Fig. 1b,c,e,f,h,i,k,l and Figs 2, D1) for both within- and between-plot levels and for all trees and three size classes. An increasing species turnover but little-to-no variation in functional turnover could be resulted from stochastic processes due to the independence between colonization and species traits. Alternatively, this pattern may also be created by random arrival order and timing of species with similar traits (priority effects) under a constant environmental filtering^24^,^25^. Furthermore, functional and phylogenetic turnover did not significantly deviated from random expectations (Fig. 1b,c,e,f,h,i,k,l and Figs 2, D1), supporting the dominance of stochastic processes but rejecting the possibility of a priority effect under constant environmental filtering.

Our findings contradicted the general expectation from recent studies that deterministic processes dominate the forest succession^17^,^18^,^19^. There are some potential reasons for only little-to-no change in functional and phylogenetic turnover along the disturbance gradient. First, it is possible to miss the full gradient in this study as we did not include early successional forests before canopy closure and very old forests where succession may last for several centuries into our analyses^6^,^26^. Next, resprouting from both cut trees and shrubs may have major impacts on species composition in subtropical forests^27^,^28^,^29^. Some species such as *Schima superba*, *Castanopsis fargesii*, *Camellia fraterna*, and *Eurya rubiginosa*, could even colonize the forest from early to late successional stages. Their resprouting could impact on late-arriving species as demonstrated by Fukami *et al*.^15^. Finally, it is possible that random immigration may play an important role in community assembly over succession^6^. Therefore, it is likely that both initial community composition and random immigration may play important roles in community assembly. The mechanisms underlying random immigration may be resulted from weak trait-environment relationship in GNNR^30^,^31^. Letten *et al*.^32^ also found that phylogenetic and functional dissimilarity did not increase during the temporal succession in a fire-prone heathland in southeast Australia. They interpreted the stochastic functional and phylogenetic turnover as being due to changes in relative fitness of different traits through time, another possible mechanism underlying random immigration in GNNR.

### The weak evidence of deterministic processes

There were limited patterns showing significant changes of functional and phylogenetic turnover along disturbance gradient at two spatial scales and three size classes (Fig. E1e,h,i,n,p,q,E2i,p,r). The significant increase in phylogenetic turnover over time for smaller size classes (Fig. E1h,n,p,q,E2p) indicated higher level of phylogenetic relatedness within subplots at late-successional stage than at early stage. This suggested an increase of the variation in habitat conditions during post-disturbance recovery^4^. In contrast, the significant decline in phylogenetic turnover for larger dbh size classes (Fig. E1i,E2i,r) indicated lower level of phylogenetic relatedness within subplots at late-successional stage than early stage. This suggested the importance of interspecific interactions for larger size classes. However, the inconsistence between patterns of phylogenetic and functional turnover together with weak phylogenetic signal in traits suggested only weak evidences for above-described deterministic processes. These results were similar to the findings of Fukami *et al*.^15^ that increasing species turnover with decreasing functional turnover was likely produced by an interaction between priority effect and decreasing variation in environmental conditions. Purschke *et al*.^19^ found simultaneous declines in species, phylogenetic, and functional turnover with a decreasing variation in environmental conditions. Our result for the role of deterministic processes, was also consistent with those from analyses of phylogenetic and functional alpha diversity of the same chronosequence^33^.

The significant effect of the interaction term of disturbance regimes and distance of habitat conditions in most functional and phylogenetic dissimilarity models (e.g. Tables F1–F2) also provided evidence of deterministic processes. This result together with insignificant functional and phylogenetic turnover suggests an alternative explanation to the dominance of stochastic assembly, that the deterministic processes in habitats of different disturbance regimes may follow different trajectories and exhibit an overall stochastic assembly. This result also partly supported the importance of trait filtering during succession in GNNR^34^.

Despite a weak phylogenetic signal, phylogenetic turnover showed coherent patterns with functional turnover, suggesting trait conservatism in our study system. However, the hypothesis using community phylogenetic structure to infer the assembly processes is currently under debate^35^. As Mayfield and Levine^35^ noted, the hypothesis could be reinterpreted by Chesson’s framework how niche and competitive ability difference could influence species coexistence^36^, and evidence has recently begun to emerge from controlled experiments^37^,^38^,^39^. In this study, when functional and phylogenetic distances are correlated with competitive ability differences that generally exceed niche difference in a species pool, our inference of ecological processes from patterns of beta diversity should be robust. However, it could be difficult to infer ecological processes in this study when niche differences exceed competitive ability difference or phylogenetic and functional distances are correlated with niche differences. Our inference of ecological processes in this subtropical forest may be valid because the competitive ability differences likely exceed niche differences for a large proportion of species pool in such a heterogeneous landscape.

## Conclusions

Disentangling the relative roles of deterministic and stochastic processes in shaping community dynamics over succession remains a major challenge in community ecology. Previous studies often hold that deterministic processes such as environmental filtering and competition dominate forest succession, leading to a transition from functional and phylogenetic clustering at earlier successional stages to functional and phylogenetic dispersion at later successional stages^17^,^18^. Contrary to this expectation, three lines of evidence from species, phylogenetic, and functional turnover revealed that community dynamics appear to be generally dominated by stochastic processes in this subtropical forest. Stochastic processes such as priority effect and random immigration, may play important roles in community assembly over succession. On the other hand, our finding of the weak evidence for deterministic processes may suggest an alternative explanation that deterministic processes in habitats of different disturbance regimes may follow different trajectories and exhibit an overall stochastic assembly. The dominance of stochastic processes over succession have important implications for subtropical forest management that the outcome of restoration would be largely unpredictable and difficult to control.

## Methods

### Study location and secondary forest plots

This study was conducted in the Gutianshan National Nature Reserve (GNNR) in the western part of Zhejiang Province, southeastern China (Fig. A1). The climate of GNNR is seasonal, and the vegetation represents a typical subtropical evergreen broad-leaved forest^40^. Detailed descriptions of climate, topography and flora can be found in Bruelheide *et al*.^6^ and Feng *et al*.^33^.

Forest plots were randomly selected and stratified by disturbance regimes in the GNNR. Three disturbance regimes of forests were estimated based on knowledge of local logging events, forest physiognomy (Fig. A3) and a dendrochronological study on the forest age^41^: 1) twice-cut forest: naturally restored forests after clear-cutting 50 years ago and selective cutting for forest tending 20 years ago (plots 1–3); 2) once-cut forest: naturally restored forests after clear-cutting 50 years ago (plots 4–6), and 3) old growth forest: forests without human disturbance for more than 100 years (plots 7–9). Three 1-ha plots (100 m in north-south direction by 100 m in west-east direction) were randomly selected within each disturbance regime, with a total of nine 1-ha forest plots established in the reserve in 2008^33^ (see details on diversity and density in Appendix A).

All trees with diameter at breast height (dbh) ≥ 1 cm were tagged, identified and spatially mapped. Topographical variables were measured during the plot establishment in 2008. A total of 58,401 tree individuals belonging to 51 families and 183 species were recorded in these plots. All nomenclature in the datasets follows the Flora of China^42^. For the present study, the trees were grouped into subplots of 10 × 10 and 20 × 20 m^2^ in size, which allows for division of each plot into subplots of equal sizes.

Elevations for all of the intersections of the 20 m grid (324 intersections) were measured, and some of the intersections of the 10 m, 5 m or smaller grids (112 intersections) were also measured in some areas of rugged topography. Maximum elevation of the 9 plots ranged from 372.6 m to 772.9 m above sea level. Four topographic variables were calculated from elevation measurements: mean elevation, terrain convexity, slope and aspect as proxy variables to comprehensively portray the overall quality of habitat^40^,^43^.

### Phylogenetic tree construction

A molecular community phylogeny was reconstructed from DNA barcode for the tree community representing 177 species in the plots, and other 6 species were excluded from the analyses in this study. Leaf tissue was collected from three individuals for each species at GNNR and desiccated with silica gel. Total DNA was extracted from samples of leaf tissue using a standard CTAB protocol^44^. Three commonly used plant chloroplast DNA barcode loci (*rbcL*, *matK* and *trnH-psbA*) were amplified and sequenced using methods from Pei *et al*.^45^. The *matK* and *rbcL* loci were then globally aligned and the *trnH-psbA* sequences were aligned within orders using MUSCLE^46^, and three loci were assembled into a DNA supermatrix. Three-partition GTR + GAMMA models were applied to the three regions separately, and a maximum likelihood algorithm was subsequently used to reconstruct community phylogenies for the plots using RAxML^47^. A rapid bootstrapping analysis with 1000 replicates was conducted to assess the percentage support for each node. Finally, an ultrametric tree was obtained using the non-parametric rate smoothing approach in the r8s software package^48^.

### Functional trait collection

We measured a total of six traits to quantitatively represent several important axes of plant functional strategy. Specific leaf area (SLA), leaf phosphorus content (LPC) and leaf nitrogen content (LNC) were measured to represent leaf economics spectrum^49^. Wood density (WD) was measured to represent wood economics spectrum^50^. Maximum height (MH) was used to represent adult light niche and competitive ability with neighbours^49^, and leaf area (LA) was measured to represent a balance between area deployed for light capture and leaf temperatures^49^. The trait collection protocols followed Cornelissen *et al*.^51^ with the exception of WD which followed the protocols of Wright *et al*.^50^.

Leaf area, SLA, LPC and LNC were measured using at least ten mature leaves collected from the tallest portion of the canopies of 5–10 of the largest individuals of each species randomly selected from the plots following the standard protocol^51^. The WD was calculated by collecting wood samples from 5 to 10 individuals for each species in the area surrounding the plots using methods described in Wright *et al*.^50^. The MH values were estimated using values reported in the Flora of China^42^. For each trait, a dendrogram was constructed using UPGMA clustering based on a Euclidean distance matrix representing interspecific trait similarity with species-level mean trait values of each species. In order to obtain a composite measure of functional similarity between species, a principal component analysis (PCA) was performed on a log-transformed and standardized trait data. Subsequently, a dendrogram of all traits was constructed by using UPGMA clustering based on a Euclidean distance of PCA axes^52^.

### Phylogenetic signal in functional traits

To examine the extent to which the phylogenetic relatedness between species reflects ecological similarity, we used the *K* statistic of Blomberg *et al*.^23^ to quantify the phylogenetic signal of available functional traits in this study. The *K* statistic provides a comparison between observed and expected levels of a phylogenetic signal under an assumption of Brownian motion trait evolution for a given phylogenetic tree^23^. Values of *K* < 1 (or *K* > 1) indicate less (or more) phylogenetic signal in trait data than expected under Brownian motion trait evolution. We assessed the significance of the phylogenetic signal by randomly shuffling tip species 999 times and calculating 95% confidence intervals.

### Quantifying the spatial turnover in species, phylogenetic and functional diversity

We quantified species beta diversity by calculating the Chao-Jaccard dissimilarity index^53^. Chao *et al*.^53^ showed that Jaccard’s dissimilarity index is systematically biased towards underestimation of similarity in hyperdiverse communities that are undersampled. The Chao-Jaccard dissimilarity index is an abundance-based dissimilarity index that incorporates the estimates of the numbers of unseen species and minimizes sampling biases^53^.

We used an index of abundance-weighted spatial partitioning of pairwise phylogenetic distance within- and between communities to measure phylogenetic beta diversity (*B*_*st*_) and functional beta diversity (*τ*_*st*_) between two communities. *Bst* and *τ*_*st*_ was calculated as^54^,^55^:


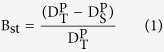



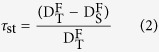


where 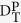 and 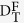
were the mean abundance-weighted phylogenetic and functional distance between community *k* and *l* respectively. 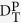 and 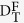 were measured as:


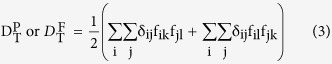


where 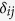 was phylogenetic or functional distance between species *i* and *j*, *f*_*ik*_ was the relative abundance of species *i* in community *k* and *f*_*jl*_ was the relative abundance of species *j* in communities *l*. 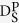 and 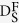 were the mean abundance-weighted phylogenetic and functional distance within community *k* and *l* respectively. Similarly, 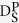
and 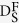 are measured as:


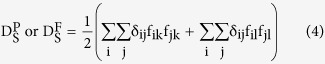


### Within-plot and between-plot dissimilarity analysis

In order to determine the effect of disturbance regimes on dissimilarity within each plot, we calculated pairwise species, phylogenetic and functional dissimilarity between all subplot (10 × 10 or 20 × 20 m^2^) pairs within each plot using Chao-Jaccard’s dissimilarity, *B*_*st*_ and *τ*_*st*_. We then used a linear mixed model to determine the effect of disturbance regimes on within-plot dissimilarity.

Studying the effect of disturbance regimes on forest succession has been criticized due to the unrealistic assumption that plots in each disturbance regime differ only in disturbance regime and have homogeneous habitat conditions^13^,^56^. We therefore removed the effect of within-plot (i.e. subplot-to-subplot) habitat difference by including the logarithm-transformed Euclidean distance of habitat variables and the logarithm-transformed spatial distance between subplots in the model. The distance of habitat conditions between each subplot pair was calculated as Euclidean distance of topographic variables after mean elevation, slope, and convexity were standardized to a zero mean and unit variance, and aspect was sine- and cosine-transformed as easting and northing. We included the mean elevation of each plot to control for the effect of elevational difference on diversity turnover at the plot scale due to elevational differences among plots (as shown in Fig. A2).

As forest plots were not equidistantly located in the reserve (Fig. A1), the logarithm-transformed spatial distance between each pair of subplots was also included as fixed effect variable in the model to remove the effect of physical proximity and other spatially autocorrelated unmeasured habitat factors such as soil nutrients. We used disturbance regimes (as a categorical variable), logarithm-transformed habitat distance and their interactions, mean elevation of plot, and logarithm-transformed spatial distance among subplots as fixed effect variables in this model. The pairwise diversity dissimilarity between subplots (level 1) was nested within plots (level 2), and each plot was, in turn, nested within a category of disturbance regime (level 3). Therefore, we included a plot-specific variable and a disturbance regime-specific variable, as random effect variables to remove the effect of plot and disturbance regime-specific conditions and the autocorrelation of spatial turnover within the same plot and the same disturbance regimes. We calculated the spatial correlogram of model residual against distance classes to check spatial autocorrelation of residuals of all models (Appendix C). Similar to the within-plot dissimilarity analysis, we used a linear mixed model to determine the effect of disturbance regime on phylogenetic and functional dissimilarity of subplots pairs between plots within each disturbance regime.

The significance of fixed effect variables were computed using F-tests for linear mixed models based on Satterthwaite’s approximation for denominator degrees of freedom^57^, and all insignificant terms of fixed effect variables were removed. A likelihood ratio test was used to compare the one level and two level random-effect variable models. Finally, because we were interested in the difference in dissimilarity between disturbance regimes, the appropriate linear contrasts were set up and tested, controlling for multiple comparisons, and the *p*-value of multiple comparisons was adjusted using the single-step method^58^.

### Null model analysis

To examine whether species within communities were phylogenetically or functionally more (or less) than expected from a random assemblage from the pool of species in this study, we compared observed values of phylogenetic (*B*_*st*_) and functional (*τ*_*st*_) beta diversity with the null *B*_*st*_ and *τ*_*st*_ values from 999 random communities for each subplot pair. Random communities were generated by randomly shuffling the species names across the tips of phylogeny or functional dendrogram. Thus, the null model randomized the phylogenetic relatedness and functional similarity of species, while maintaining the observed species abundance, species richness, and species beta diversity in space. The above-described linear mixed model was used to control the effect of habitat difference and other potentially important factors on the observed and null *B*_*st*_ and *τ*_*st*_ values.

We also examined species, phylogenetic and functional beta diversity within each age category. We divided stems into three size classes: dbh 1 cm–5 cm, dbh 5 cm−10 cm, and dbh >10 cm to represent different regeneration classes during succession. We expected that phylogenetic and functional beta diversity changes across dbh size classes. This expectation follows because smaller stems in each plot were assumed to colonize at more recent successional stage within each plot, whereas larger stems established earlier in the process of succession.

Chao-Jaccard’s dissimilarity index was calculated using the R package ‘vegan’^59^. The *B*_*st*_ and *τ*_*st*_ values and null models of *B*_*st*_ and *τ*_*st*_ was calculated using the R package ‘spacodiR’^60^. Linear mixed models were fitted using the R package ‘lme4’^61^. The significance of fixed effect variables were computed using ‘lmerTest’ package^57^, and multiple comparisons were conducted using the ‘multcomp’ package^58^. All of the computations were conducted in R^62^.

## Additional Information

**How to cite this article**: Mi, X. *et al*. Stochastic assembly in a subtropical forest chronosequence: evidence from contrasting changes of species, phylogenetic and functional dissimilarity over succession. *Sci. Rep*. **6**, 32596; doi: 10.1038/srep32596 (2016).

## Supplementary Material

Supplementary Information
