# Supplementary material for: Stochastic assembly in a subtropical forest chronosequence: evidence from contrasting changes of species, phylogenetic and functional dissimilarity over succession
Source: Sci Rep. 2016 Sep 7;6:32596. doi: 10.1038/srep32596 (PMC5013490; doi:10.1038/srep32596)
Supplement: Supplementary Information [file srep32596-s1.pdf]

Stochastic assembly in a subtropical forest chronosequence: evidence from contrasting changes of species, phylogenetic and functional dissimilarity over succession

Xiangcheng Mi, Nathan G. Swenson, Qi Jia, Mide Rao, Gang Feng, Haibao Ren, Daniel P. Bebber, Keping Ma

Appendix A. Study area and basic description of the plots

Figure A1

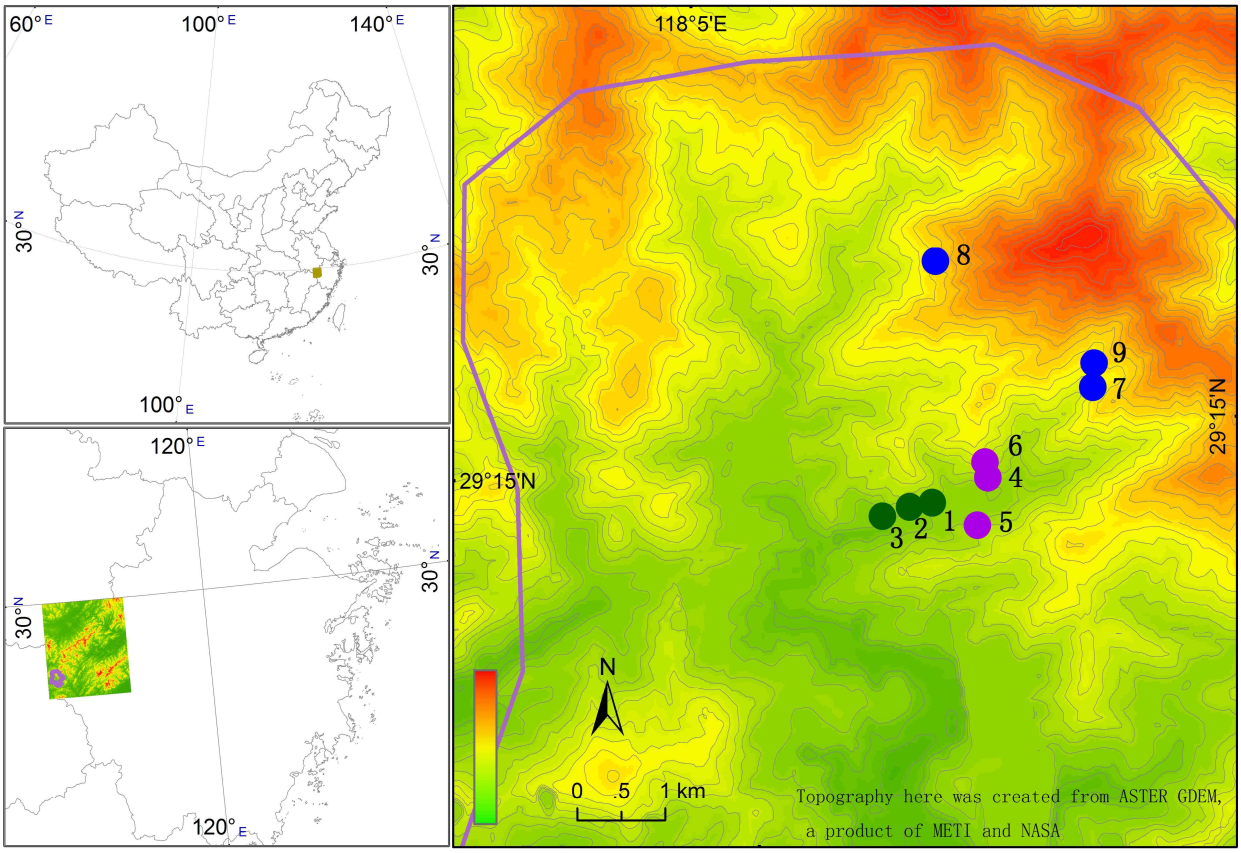

Figure A1. Locations of study area and Gutianshan National Nature Reserve (NNR). a) Zhejiang Province with Gutianshan NNR in China; b) Gutianshan NNR in Zhejiang Province; c) Gutianshan NNR with nine 1-ha plots. The purple lines in b and c are the boundary of Gutianshan NNR. Twice-cut forest: plots 1-3 have been naturally restored forests after clear-cutting 50 years ago and selective

cutting for forest tending 20 years ago. Once-cut forest: plots 4-6 have been naturally restored forests after clear-cutting 50 years ago. Old growth forest: plots 7-9 are old-growth forests without human disturbance more than 100 years (figure created from ASTER global digital elevation model owned by METI and NASA: http://www.jspacesystems.or.jp/ersdac/GDEM/E/4.html and authors' data with ArcGiS 10.0 (ESRI): www.esri.com/software/arcgis/).

Fig. A2

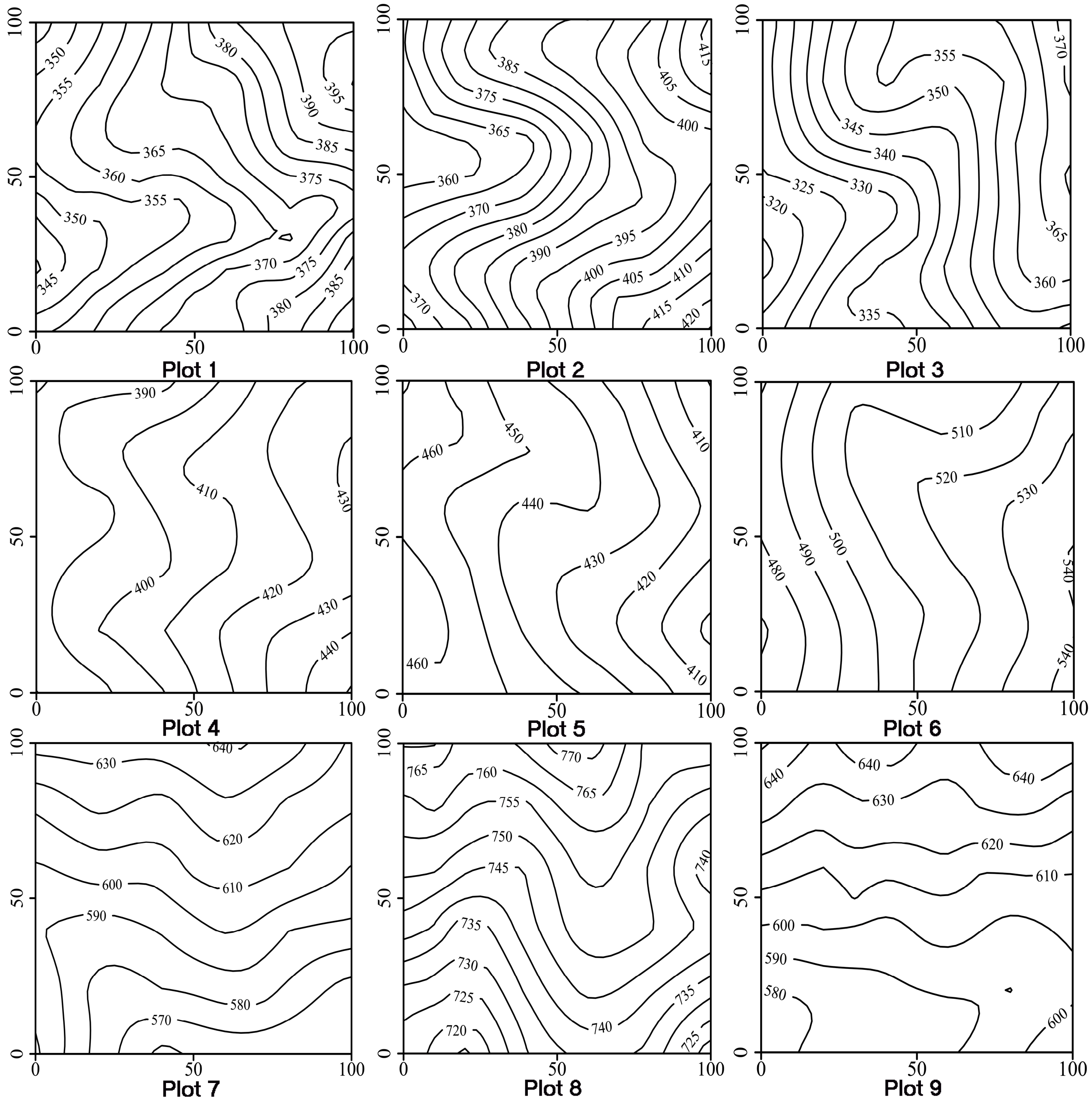

Figure A2. Topography map of 9 1-ha plots along a disturbance gradient in Gutianshan Nature Reserve. 1) twice-cut forest: plots 1-3; 2) once-cut forest: plots 4-6; 3) old growth forest: plots 7-9.

Fig. A3

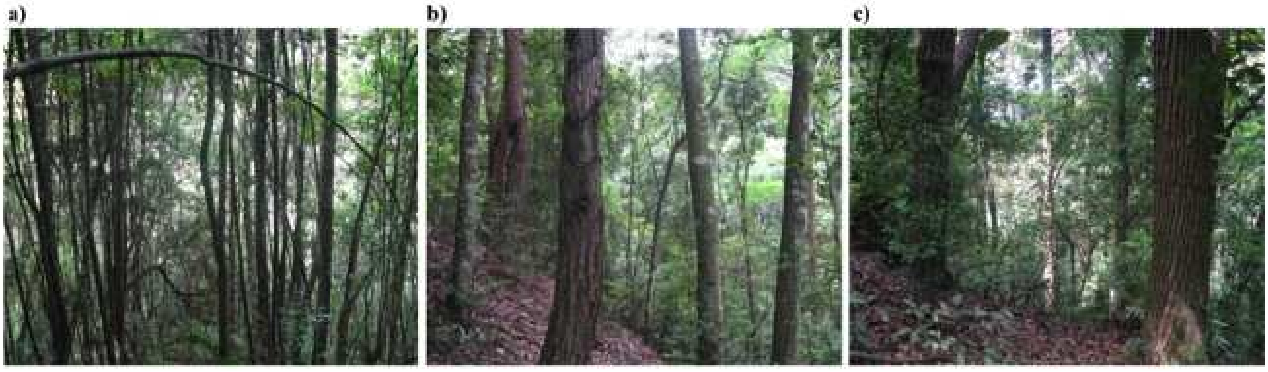

Fig. A3. Photos of forest types in this study. Each photo was used to illustrate the forest physiognomy and structure difference among a) twice-cut forest, b) once-cut forest, c) old growth forest.

Photo Credit: Xiangcheng Mi

Table A1. The description of diversity, elevation range, maximum elevation difference in each plot and density of 9 1-ha forest plots

**Plot**	**Successional stage**	**Elevation range (m)**	**Maximum elevation difference (m)**	**Species richness**	**Tree density (individuals/ha)**
**1**	I	338.9-400.3	61.4	100	11,611
**2**	I	356.1-425.5	69.4	95	10,467
**3**	I	312.9 - 372.6	59.7	85	8,323
**4**	II	377.5-451.3	73.8	105	5,303
**5**	II	396.7-472.0	75.3	123	5,501
**6**	II	468.2-544.9	76.7	87	5,138
**7**	III	556.9-643.0	86.1	114	4,678
**8**	III	713.8-772.9	59.1	89	6,724
**9**	III	575.8-652.1	76.3	118	4,381

Appendix B Phylogenetic signal analysis of available functional traits

Table B1 The phylogenetic signal analysis of available functional traits

Traits	K value	P value
**Specific leaf area (SLA)**	0.108	0.049
**Leaf area**	0.105	0.154
**Leaf phosphor content**	0.160	0.004
**Leaf nitrogen content**	0.255	0.001
**Maximum height**	0.441	0.001
**Wood density**	0.214	0.001
**Axis 1 of PCA**	0.270	0.007
**Axis 2 of PCA**	0.342	0.001

Appendix C Spatial Correlograms of residuals of linear mixed models

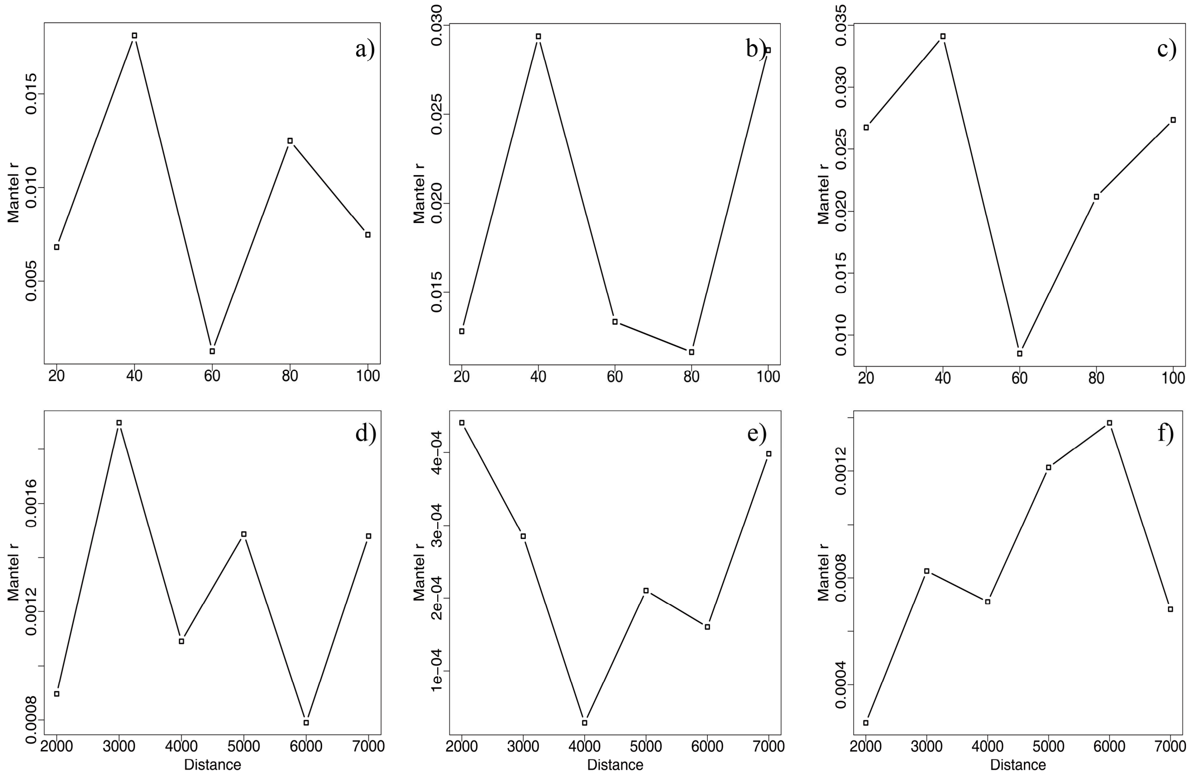

Figure C1. The spatial correlogram of residuals of linear mixed models for within-plot and between-plot beta diversity against distance classes at a scale of 20×20 m. a) is spatial correlogram of residuals of linear mixed model of species beta diversity, b) for phylogenetic beta diversity, c) for functional beta diversity of all traits at within-plot level, while d) is spatial correlogram of residuals of linear mixed model of species beta diversity, e) for phylogenetic beta diversity, f) for functional beta diversity of all traits at between-plot level. All of Mantel r in the figure is not significant ($p > 0.05$).

Appendix D. The within-plot and between-plot functional beta diversity of individual traits at scales of 10×10 m

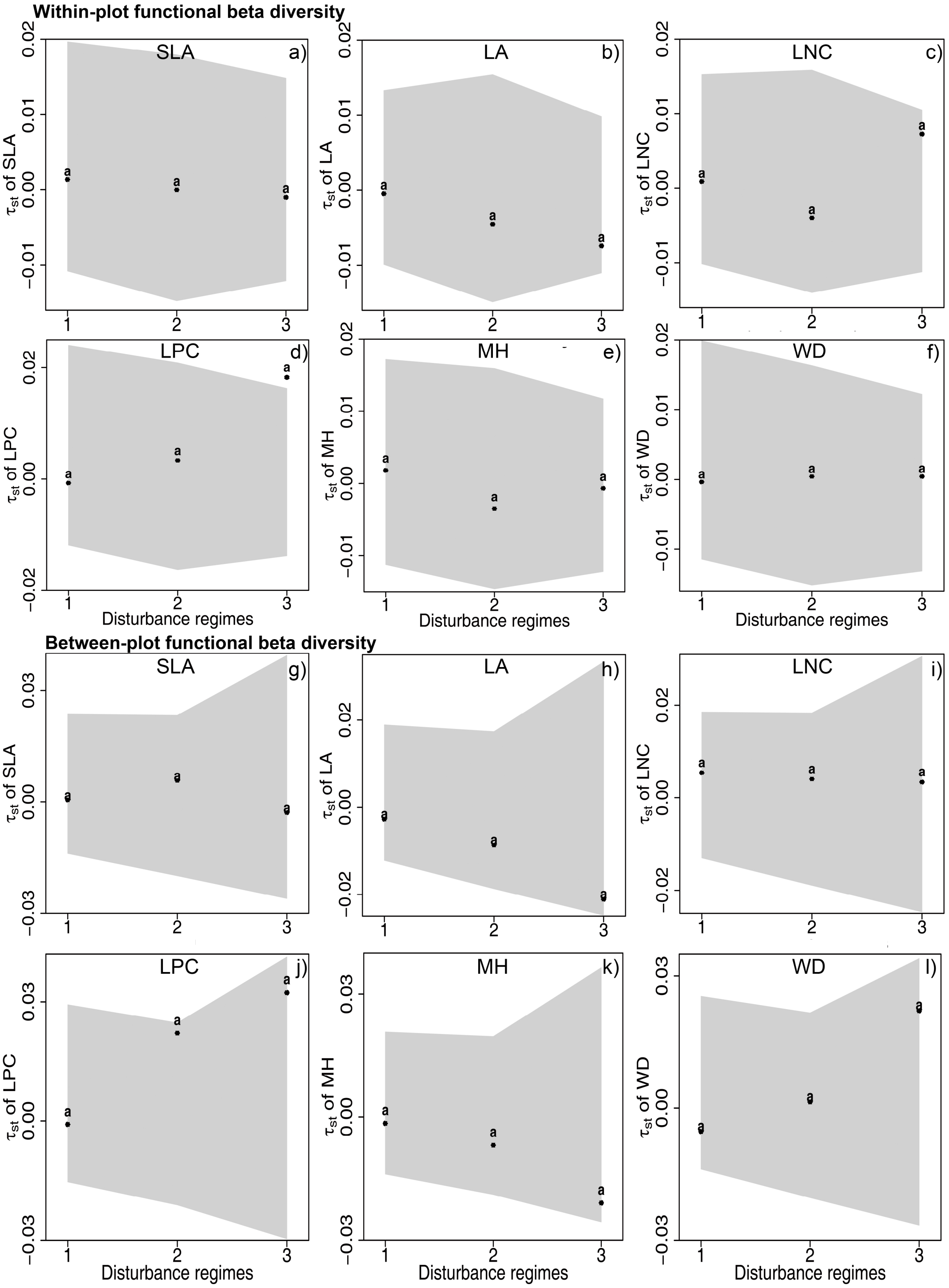

Figure D1. The within-plot (a-f) and between-plot (g-l) functional beta diversity of individual traits for all pairs of quadrats (black square, mean ± SE) within disturbance regime at a scale of 10×10 m. Different letters indicate significant differences ($P<0.05$) between the effect among disturbance regimes, and the significance of difference between disturbance regimes was calculated by multiple comparison. The grey-shaded area represent the 95%-confidence interval for the τ_{st} values from the 999 random communities. τ_{st} values inside the interval indicate phylogenetic or functional randomness, and τ_{st} values outside the interval indicate the significant phylogenetic or functional clustering or dispersion. SLA = specific leaf area, LA = leaf area, WD = wood density, LPC = leaf phosphorus content, LNC = leaf nitrogen content, MH = maximum height. Disturbance regimes: 1. twice-cut forest: clear-cutting 50 years ago and selective cutting 20 years ago, 2. once-cut forest: clear-cutting 50 years ago, 3. old growth forest: without human disturbance more than 100 years.

Appendix E Species, phylogenetic and functional beta diversity of different disturbance regimes at within-plot and between-plot levels and at different scales

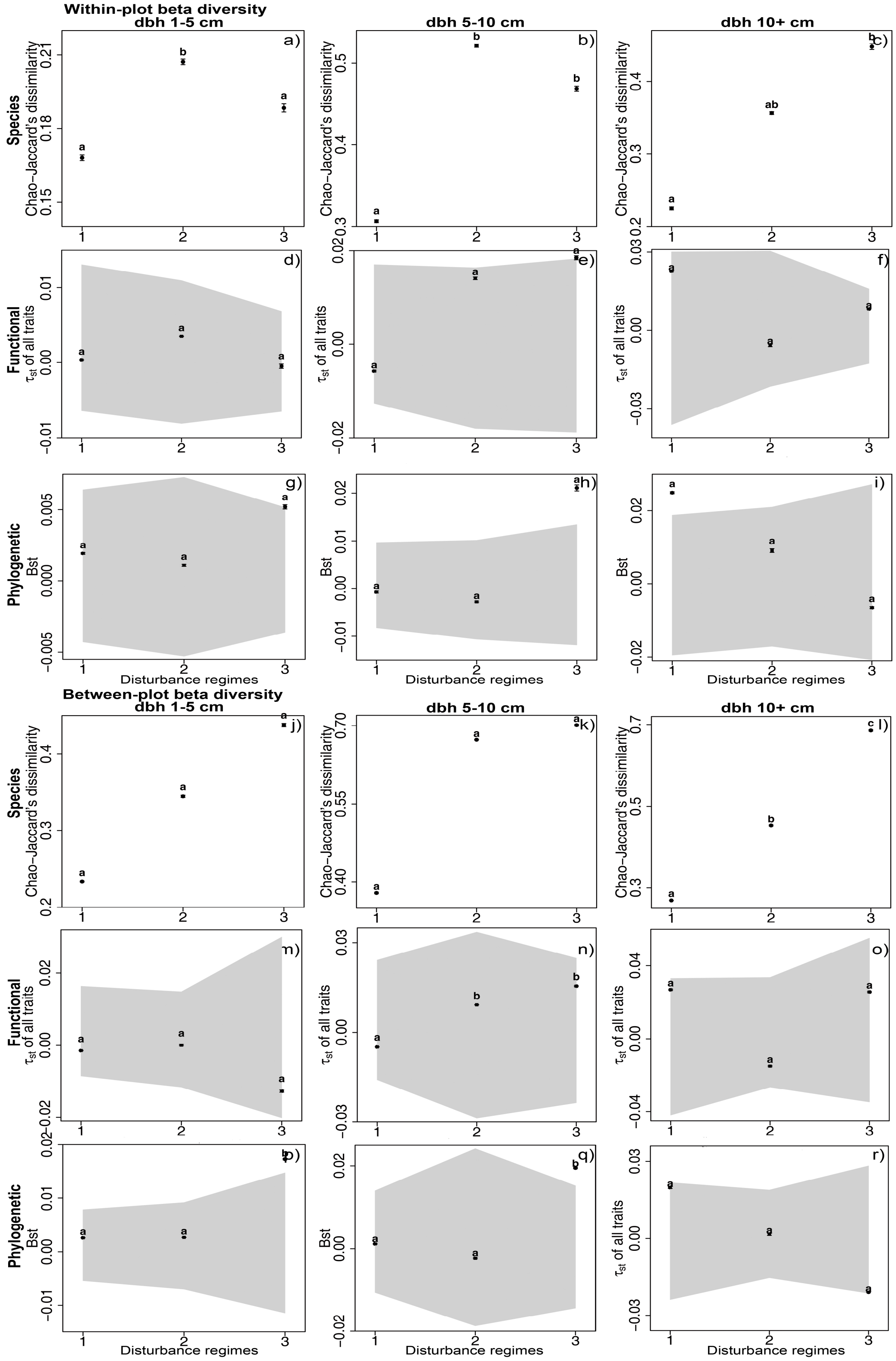

Fig. E1. Within-plot and between-plot species, phylogenetic and functional beta diversity for all pairs of quadrats (black square, mean±SE) within different disturbance regimes at a scale of 20×20 m. Different letters indicate significant differences ($P<0.05$) between disturbance regimes, and the significance of difference between disturbance regimes was calculated by multiple comparison. The grey-shaded area represent the 95%-confidence interval for τ_{st} and B_{st} values from the 999 random communities. τ_{st} and B_{st} values inside the interval indicate phylogenetic or functional randomness, and τ_{st} and B_{st} values outside the interval indicate the significant phylogenetic or functional clustering or dispersion. The right, middle and left panels showed phylogenetic beta diversity for cohorts with 1-5 cm dbh, 5-10 cm dbh, and larger than 10 cm. Disturbance regimes: 1.twice-cut forest: clear-cutting 50 years ago and selective cutting 20 years ago, 2. once-cut forest: clear-cutting 50 years ago, 3. old growth forest: without human disturbance more than 100 years.

Fig. E2

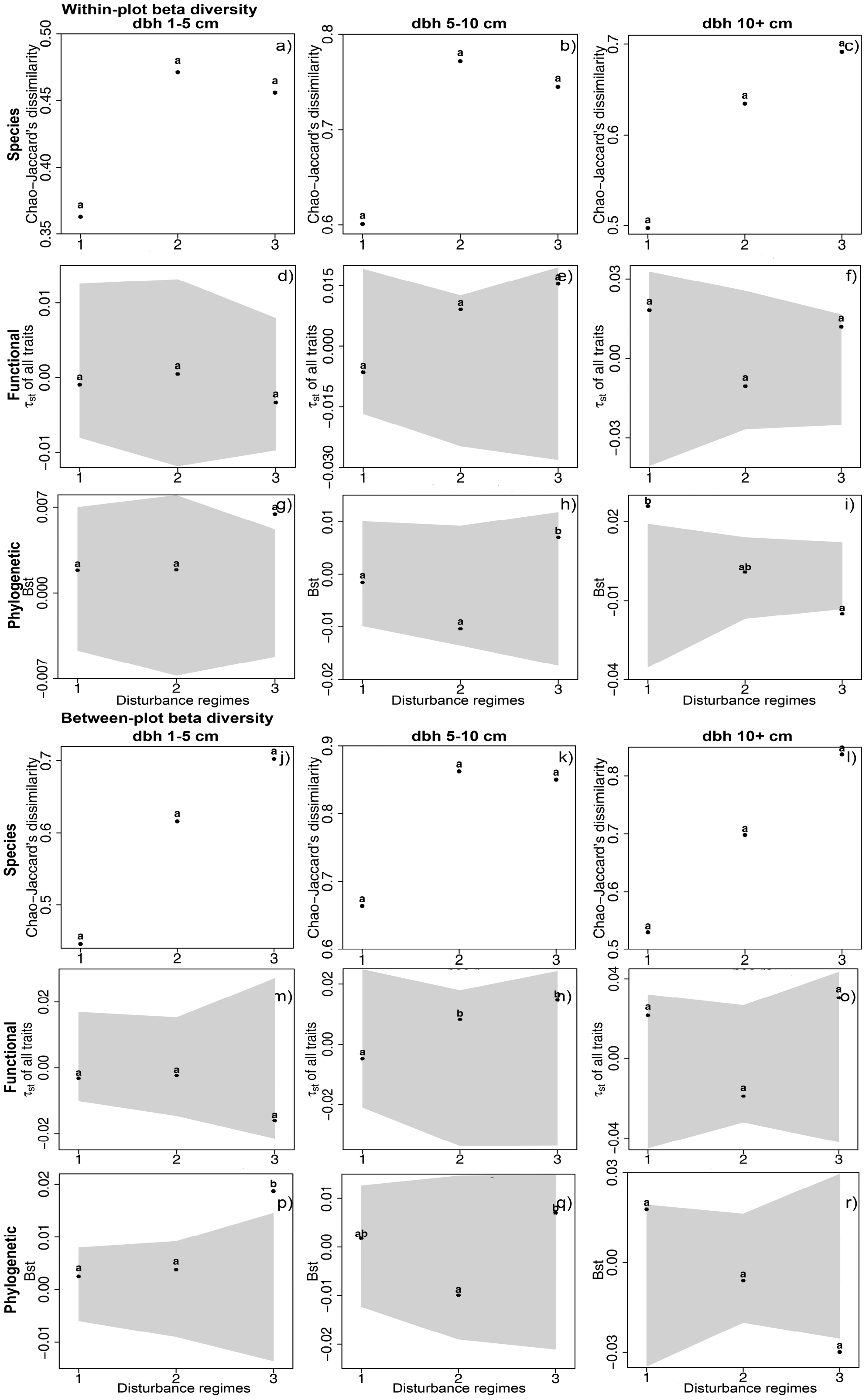
Within-plot beta diversity
dbh 1-5 cm
dbh 5-10 cm
dbh 10+ cm
Species
Chao–Jaccard's dissimilarity
Functional
τ_{st} of all traits
Phylogenetic
Bst
Disturbance regimes
Between-plot beta diversity
a) b) c) d) e) f) g) h) i) j) k) l) m) n) o) p) q) r)

Fig. E2 Within-plot (a-i) and between-plot (j-r) species, phylogenetic and functional beta diversity for all pairs of quadrats (black square, mean±SE) in different disturbance regimes at a scale of 10×10 m. Different letters indicate significant differences (P<0.05) between disturbance regimes, and the significance of difference between disturbance regimes was calculated by multiple comparison. The grey-shaded area represent the 95%-confidence interval for the τ_{st} and B_{st} values from the 999 random communities. τ_{st} and B_{st} values inside the interval indicate phylogenetic or functional randomness, and τ_{st} and B_{st} values outside the interval indicate the significant phylogenetic or functional clustering or dispersion. The right, middle and left panels showed functional beta diversity for cohorts with 1-5 cm dbh, 5-10 cm dbh, and larger than 10 cm. Disturbance regimes: 1.twice-cut forest: clear-cutting 50 years ago and selective cutting 20 years ago, 2. once-cut forest: clear-cutting 50 years ago, 3. old growth forest: without human disturbance more than 100 years.

Appendix F Effect of disturbance regime and other significant factors on species, phylogenetic and functional beta diversity

Table F1. Effect of disturbance regimes and other significant factors on species and phylogenetic beta diversity at within-plot and between-plot levels and at scale of 20×20m

Beta diversity type		Statistics				
		Predicting variables	DF1	DF2	F	P
**Within-plot beta diversity**	Chao-Jaccard dissimilarity	Disturbance regime	2.0	6.5	0.79	0.491
		Spatial distance	1.0	2686.4	142.76	<.001
		Disturbance regime×habitat distance	3.0	2687.8	7.13	<.001
	Bst	Disturbance regime	2	6.0	0.17	0.845
		habitat distance	1	2665.4	12.71	<0.01
	τ_{st}	Disturbance regime	2.0	6.85	0.584	0.583
		Disturbance regime ×habitat distance	3.0	2659.2	10.50	<.001
**Between-plot beta diversity**	Chao-Jaccard dissimilarity	Disturbance regime	2	6.4	6.47	0.029
		Habitat distance	1	5614.1	11.00	0.001
		Disturbance regime ×habitat distance	2	5614.0	28.78	<.001
	Bst	Disturbance regime	2	2.2	0.092	0.915
		Habitat distance	1	5566.6	33.26	<.001
		Spatial distance	1	19.2	15.99	<0.001
		Disturbance regime ×habitat distance	2	5562	18.02	<.001
	τ_{st}	Disturbance regime	2	6.4	0.424	0.671
		Habitat distance	1	5587.4	75.96	<.001
		Disturbance regime ×habitat distance	2	5587.3	25.08	<.001

Table F2. Effect of disturbance regimes and other significant factors on species and phylogenetic beta diversity at within-plot and between-plot levels and at scale of 10×10m

Beta diversity type		Statistics				
		Predicting variables	DF1	DF2	F	P
**Within-plot beta diversity**	Chao-Jaccard dissimilarity	Disturbance regime	2.0	6.0	1.22	0.359
		Habitat distance	1.0	44519	663.45	<.001
		Spatial distance	1.0	44516	1692.10	<.001
		Disturbance regime×habitat distance	3.0	44519	51.96	<.001
	Bst	Disturbance regime	2	6.0	0.23	0.800
		Habitat distance	1	44291	147.85	<.001
		Disturbance regime×habitat distance	2	44291	16.74	<.001
	τ_{st}	Disturbance regime	2.0	6.0	0.020	0.981
		Habitat distance	1.0	44380	22.15	<.001
**Between-plot beta diversity**	Chao-Jaccard dissimilarity	Disturbance regime	2	9.0	0.90	0.438
		Habitat distance	1	89952	738.95	<.001
		Spatial distance	1	5669	178.52	<.001
		Disturbance regime ×habitat distance	2	89951	86.19	<.001
	Bst	Disturbance regime	2	5	0.05	0.950
		Habitat distance	1	89564	262.98	<.001
		Spatial distance	1	701	62.61	<.001
		Disturbance regime ×habitat distance	3	89561	46.73	<.001
	τ_{st}	Disturbance regime	2	4	0.168	0.851
		Habitat distance	1	89702	154.01	<.001
		Spatial distance	1	217	38.42	<.001
		Disturbance regime ×habitat distance	2	89702	45.33	<.001
